# Comparison of the Therapeutic Efficacies of Topical Rivoceranib and Topical Bevacizumab in a Murine Model of Corneal Neovascularization

**DOI:** 10.3390/medicina55110729

**Published:** 2019-11-07

**Authors:** Hyeon Jeong Yoon, Je Moon Woo, Yong Sok Ji, Kyung Chul Yoon

**Affiliations:** 1 Department of Ophthalmology, Chonnam National University Medical School and Hospital, 42 Jebong-ro, Dong-gu, Gwangju 61469, Korea; yoonhyeonjeong@hanmail.net; 2 Department of Ophthalmology, Ulsan University Hospital, University of Ulsan College of Medicine, Ulsan 44033, Korea; limbus68@naver.com

**Keywords:** corneal neovascularization, rivoceranib, bevacizumab

## Abstract

*Background and Objectives:* Corneal neovasculariziation (CNV) is a serious vision-threatening complication; however, all therapeutics have their clinical limitations. The aim of this study is to investigate the efficacy of topical rivoceranib compared with topical bevacizumab in a murine model of corneal neovascularization (CNV). *Materials and Methods:* Murine CNV was induced by means of total de-epithelization and alkali burn. Mice were divided into five groups according to topical treatment: untreated control, phosphate-buffered saline (PBS), 0.1% and 0.5% rivoceranib, and 0.5% bevacizumab. CNV area and index were measured 7 and 14 days after treatment. After corneal tissues were excised at day 14, the blood and lymphatic vessels were quantified by cluster of differentiation 31 (CD31) and lymphatic vessel endothelial hyaluronan receptor 1 (LYVE1) immunofluorescence, respectively. *Results:* After 14 days, treatment groups with 0.1% and 0.5% rivoceranib and 0.5% bevacizumab showed a decrease in CNV area and index compared with the untreated and PBS groups (all *p* < 0.01). Blood and lymphatic vascularization significantly decreased in the 0.5% rivoceranib and 0.5% bevacizumab groups, as measured by CD31 and LYVE1 immunofluorescence. There was no significant difference of vascularization between the 0.5% rivoceranib and bevacizumab groups. *Conclusions:* Topical application of rivoceranib could effectively decrease CNV equivalent to topical bevacizumab in a murine model.

## 1. Introduction

Corneal neovascularization (CNV) is ingrowth of new vessels from the limbus, which can cause visual loss due to accompanying scarring and lipid deposition [1,2]. It results from acute or chronic corneal inflammation, limbal stem cell deficiency, and corneal ischemia [1,2]. Corneal angiogenesis is initiated by the imbalance between angiogenic and anti-angiogenic factors [2,3]. Angiogenic factors, including vascular endothelial growth factor (VEGF), hypoxia-inducible factor (HIF), matrix metalloproteinases (MMPs), platelet-derived growth factor (PDGF), and basic fibroblast growth factor (FGF) are upregulated during corneal angiogenesis, whereas the anti-angiogenic factors are downregulated [2,3]. 

Among these, VEGF is a key regulator in angiogenesis and is upregulated in inflammatory corneal diseases associated with CNV [4,5,6]. The VEGF family comprises several forms of VEGF and VEGF receptors. Several studies have focused on VEGF-A and its receptor, namely, VEGF receptor 1 and 2 (VEGFR-1 and VEGFR-2), which play major roles in the physiology of angiogenesis. In particular, VEGFR-2 is more potent than VEGFR-1, and mediates almost all the cellular responses to VEGF in CNV [4,5,6,7]. HIF, a factor induced in hypoxic condition, activates a signaling pathway that up-regulates VEGF expression [8,9]. MMPs, upregulated by VEGF, act upon endothelial cells in the limbal vascular plexus and stimulate blood vessel formation [2,8].

The treatments for CNV include medications, such as steroids or angiogenesis inhibitors, laser photocoagulation, and surgeries such as limbal stem cell transplantation and keratolimbal allograft; all of these have clinical limitations [1,4,6,10]. Anti-VEGF agents, including bevacizumab, ranibizumab, and aflibercept, are most commonly used to treat many ocular diseases such as retinal neovascularization in age-related macular degeneration (ARMD), diabetic retinopathy, and neovascular glaucoma [11]. VEGF has been studied as a main target in ophthalmology, and recently a new anti-VEGF agent, brolucizumab, has received FDA approval for ARMD [11]. These anti-VEGF agents have been widely investigated for CNV [6,12,13,14,15,16]. 

However, multiple compensatory angiogenic factors/signaling pathways could occur during anti-VEGF stress [9]. PDGF initiates various downstream signaling events by recruiting SH2 domain-containing molecules such as ERK kinase, PI3K, FAK, and mTOR [8,9]. FGF, another cytokine that plays an important role in angiogenesis, could involve angiogenetic signaling by molecules including PI3K, PLC-r, and RAS [9,17].

Rivoceranib, a novel and selective potent VEGFR-2 tyrosine kinase inhibitor (TKI), can restrain several signaling pathways of VEGFR-2 such as the Raf/MEK/Erk, p38-MAPK, and PI3K/AKT/mTOR pathways, which results in vascular angiogenesis [18,19,20]. Recently, several studies have shown that rivoceranib could directly inhibit the PI3K/AKT signaling pathway, which was associated with a VEGF-independent compensatory pathway in angiogenesis [19,21].

The effectiveness of rivoceranib has already been proven in metastatic solid cancer, including gastric, colorectal, hepatocellular, and lung cancer [18,20,22]. Thus, we thought that rivoceranib could be a potential therapeutic agent in CNV. There has only been one study about the effects of rivoceranib on CNV [23]. Additionally, several studies on the inhibition of tyrosine kinase pathway for CNV have focused on multi-targeted TKIs [14,24,25,26,27,28]. In this study, we compared the efficacy of topical rivoceranib to that of topical bevacizumab in the murine model of CNV.

## 2. Materials and Methods

### 2.1. Preparation of Drug

Rivoceranib (apatinib mesylate) in powder form (CAS number 1218779-45-9; LSK biopharma, Salt Lake City, UT, USA) was dissolved in sodium carboxymethyl cellulose (Sigma-Aldrich, Darmstadt, Germany) to concentrations of 1 mg/mL (0.1%) and 5 mg/mL (0.5%). Bevacizumab (0.5%) (Avastin; Roche, Welwyn Garden City, UK), used as the positive control, was prepared by dilution with normal saline.

### 2.2. Mouse Model of Corneal Neovascularization

The animal research protocol was approved by the Chonnam National University Medical School Research Institutional Animal Care and Use Committee (CNU IACUC-H-2018-43, on 30 July 2018). Maintenance of animals and all in vivo experiments were performed in accordance with the Association for Research in Vision and Ophthalmology Statement for the Use of Animals in Ophthalmic and Vision Research. 

Male C57BL6/N mice, aged 6 to 8 weeks, were used in the following experiments. CNV was induced by de-epithelization and alkali burn [29,30]. Briefly, the eyes were anesthetized by topical 0.5% proparacaine. A paper disc (3 mm in diameter) soaked with 0.1 N NaOH was placed on the ocular surface of the right eye for 10 seconds, after which the eye was washed with 15 mL normal saline solution. After alkali burn, the entire corneal epithelium, parallel to the limbus, was scraped with a corneal knife. Moxifloxacin was applied three times a day for the first three days to prevent infection.

The mice were randomly divided into five groups as follows: (1) the untreated (UT) group, comprising control CNV mice that received no eyedrops; (2) CNV mice treated with phosphate-buffered saline (PBS); (3) CNV mice treated with 0.1% rivoceranib; (4) CNV mice treated with 0.5% rivoceranib; and (5) positive control, comprising CNV mice treated with 0.5% bevacizumab. Eye drops (2 μL) were topically applied to the right eye of the mice three times a day (at 8 a.m., 12 p.m., and 5 p.m.). Each group consisted of six animals and a total of 36 mice were used per one experiment. Three sets of experiments were performed.

### 2.3. Clinical Measurement of CNV

The clinical parameters of all mice were examined at 7 and 14 days after treatment. All the mice were photographed at a magnification of 40×. The area of CNV was calculated using the Robert model: S = C ÷ 12 × 3.1416 × (R^2^ − (R − L)^2^), where C is the time of the circumference of the cornea that accumulates for the development of the new blood vessel, R is the corneal radius, and L is the length of CNV from the corneal limbus to the center of cornea [31,32]. The CNV index was calculated as the difference between the total corneal area and the avascularized area, divided by the total corneal area. 

### 2.4. Immunohistochemical Measurement of CNV

After measuring the clinical parameters, the mice were euthanized using an intraperitoneal overdose of pentobarbital and corneas were excised from the limbus and subsequently stabilized in formaldehyde. The corneas were embedded in paraffin blocks and immunostained as previously described using fluorescein isothiocyanate (FITC)-conjugated rat anti-CD31 (blood vessel marker; BioLegend, San Diego, CA, USA) and Cy3-conjugated goat anti-LYVE1 (lymphatic marker; AbCam, Cambridge, UK) [30]. The corneas were counterstained with 4’,6-diamidino-2-phenylindole (DAPI, Vector Laboratories, Inc., Burlingame, CA, USA). The corneas were rinsed in PBS and flat-mounted on slides using a mounting medium and then stored at 4 °C in the dark until subsequent analysis via fluorescent microscope (Leica CTR5500; Leica Microsystems, Wetzler, Germany) at a magnification of 25×. Digital pictures were analyzed using ImageJ software version 1.8.0. (National Institutes of Health, Bethesda, MD, USA), and the area of CNV was encircled by drawing. The percentage of CNV area was calculated.

### 2.5. Statistical Analysis 

All the statistical analyses were performed using SPSS Statistics for Windows, version 18.0 (SPSS Inc., Chicago, IL, USA). The data are represented as median (interquartile range). A Mann-Whitney U test was used for comparing each group. A *p* value of less than 0.05 was considered to have statistical significance. Statistical significance was determined as *p* < 0.05, with differences corrected by the Benjamini-Hochberg procedure using false discovery rates of 0.25.

## 3. Results

### 3.1. CNV Area

There were no statistically significant differences in the CNV area among the groups at baseline (data not shown). Seven days after treatment, the CNV area of the 0.1% rivoceranib (63.05 (4.12) mm^2^), 0.5% rivoceranib (55.21 (9.34) mm^2^), and 0.5% bevacizumab (56.05 (3.90) mm^2^) groups were significantly decreased compared with the UT control (74.68 (5.03) mm^2^; all *p* < 0.01) and PBS (72.86 (5.02) mm^2^; *p* = 0.02, *p* < 0.01, and *p* < 0.01, respectively) treated groups.

**Figure 1:**
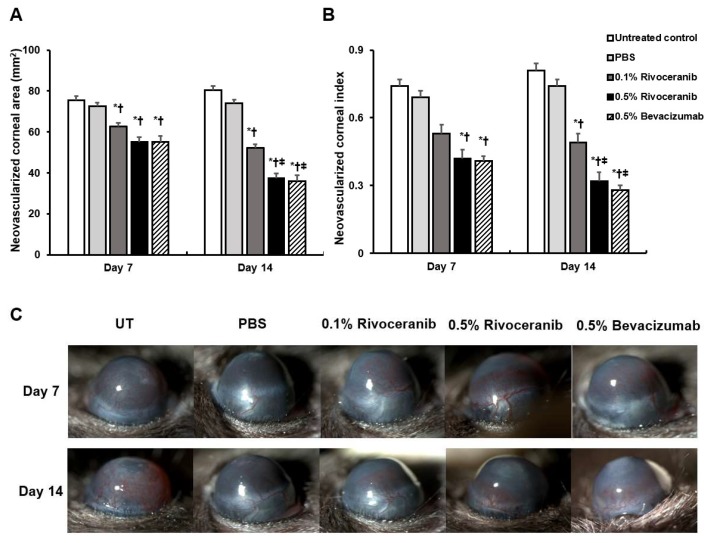
Comparison of the (**a**) area of corneal neovasculariziation (CNV) and (**b**) CNV index in the untreated (UT) control group, phosphate-buffered saline (PBS) group, and groups treated with 0.1% rivoceranib, 0.5% rivoceranib, and 0.5% bevacizumab. (**c**) Representative images of the groups. * *p* < 0.05 compared with the control; ^†^ *p* < 0.05 compared with the PBS group; ^‡^ *p* < 0.05 compared with the group treated with 0.1% rivoceranib.

At 14 days, the mean CNV area was significantly decreased to 51.91 (3.64) mm^2^, 38.29 (5.44) mm^2^, and 35.88 (10.81) mm^2^ in the 0.1% and 0.5% rivoceranib, and 0.5% bevacizumab compared with the control (80.83 (2.51) mm^2^; all *p* < 0.01) and PBS (74.51 (3.56) mm^2^; all *p* < 0.01) groups. The 0.5% rivoceranib and 0.5% bevacizumab groups showed a smaller CNV area at 14 days after treatment than the 0.1% rivoceranib group (both *p* < 0.01). There was no significant difference in the CNV area between the 0.5% rivoceranib and bevacizumab groups at 7 and 14 days (Figure 1a).

### 3.2. CNV Index

There were no statistically significant differences in the CNV area among the groups at baseline (data not shown). Seven days after treatment, the CNV index of the UT, PBS, 0.1% rivoceranib, 0.5% rivoceranib, and 0.5% bevacizumab groups was 0.72 (0.13), 0.69 (0.10), 0.55 (0.14), 0.41 (0.15), and 0.41 (0.04), respectively. The 0.5% rivoceranib and 0.5% bevacizumab treatment groups showed a significant decreased CNV index compared with the UT and PBS groups (all *p* < 0.01).

At 14 days after treatment, the CNV index of the UT and PBS groups was 0.84 (0.12), and 0.75 (0.11), respectively. The mean CNV index was significantly decreased in the 0.1% rivoceranib (0.50 (0.12)), 0.5% rivoceranib (0.30 (0.14)), and 0.5% bevacizumab (0.28 (0.07)) groups compared with the UT and PBS groups (all *p* < 0.01). Moreover, the 0.5% rivoceranib and 0.5% bevacizumab groups had a smaller CNV index than the 0.1% rivoceranib group at 14 days after treatment (both *p* < 0.01). No significant difference was shown between the 0.5% rivoceranib and bevacizumab groups with respect to the CNV index after 7 and 14 days of treatment (Figure 1b).

### 3.3. Immunofluorescent Staining of Blood Vessels 

**Figure 2:**
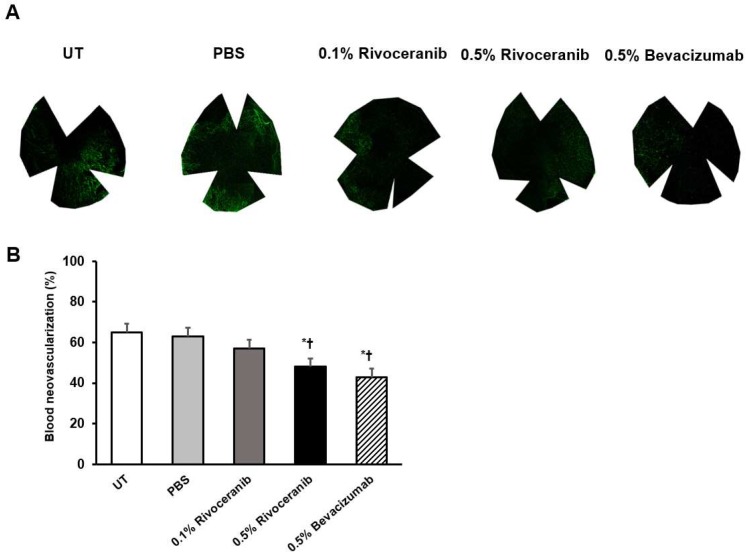
Percentage of blood neovascularization as measured by immunofluorescence staining of CD31. (**a**) Representative images of the UT and PBS groups and groups treated with 0.1% rivoceranib, 0.5% rivoceranib, and 0.5% bevacizumab. (**b**) The percentages of blood neovascularization are compared graphically. * *p* < 0.05 compared with the control; ^†^ *p* < 0.05 compared with the PBS group.

The mean percentage of blood vascularization, stained by CD31, was 65.0 (2.5)% in the control, 60.0 (5.0)% in the PBS, 55.0 (7.5)% in the 0.1% rivoceranib, 50.0 (2.5)% in the 0.5% rivoceranib, and 45.0 (2.5)% in the 0.5% bevacizumab groups, respectively. The percentage of blood vascularization was lower in the 0.5% rivoceranib and 0.5% bevacizumab groups than in the UT (*p* = 0.02 and *p* < 0.01, respectively) and PBS groups (*p* = 0.03 and *p* < 0.01, respectively). There was no significant difference in the percentage of blood vascularization between the 0.5% rivoceranib and 0.5% bevacizumab groups (Figure 2).

### 3.4. Immunofluorescent Staining of Lymphatic Vessels 

**Figure 3:**
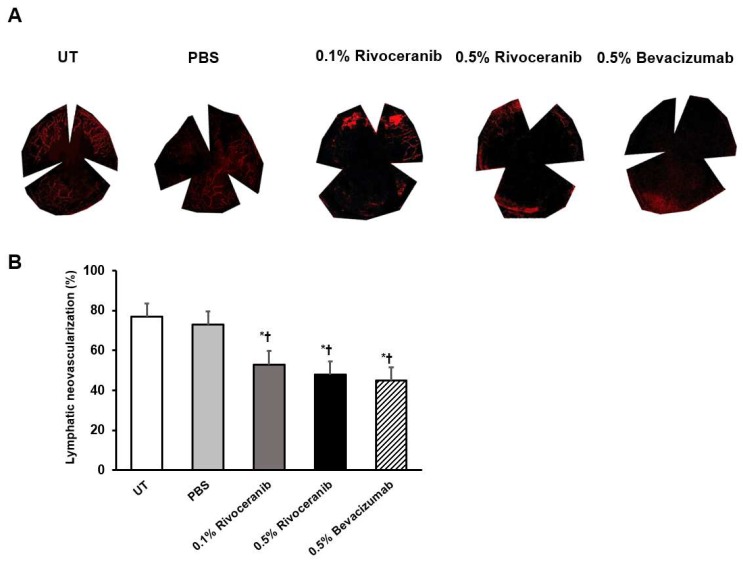
Percentage of lymphatic neovascularization as measured by immunofluorescence staining of LYVE1. (**a**) Representative images of the UT and PBS groups and groups treated with 0.1% rivoceranib, 0.5% rivoceranib, and 0.5% bevacizumab. (**b**) The percentages of lymphatic neovascularization are compared graphically. * *p* < 0.05 compared with the control; ^†^ *p* < 0.05 compared with the PBS group.

The mean percentage of lymphatic vascularization stained by LYVE1 was 75.0 (2.5)%, 75.0 (2.5)%, 50.0 (5.0)%, 50.0 (7.5)%, and 45.0 (5.0)% in the UT, PBS, 0.1% and 0.5% rivoceranib, and 0.5% bevacizumab groups, respectively. Lymphatic vascularization was decreased in the 0.1% and 0.5% rivoceranib, and 0.5% bevacizumab groups compared with the UT and PBS groups (all *p* < 0.01). There was no significant difference in the percentage of lymphatic vascularization between the 0.5% rivoceranib and 0.5% bevacizumab groups (Figure 3).

## 4. Discussion

CNV is a serious vision-threatening complication [1,4]. Angiogenesis is initiated when the balance between angiogenic and anti-angiogenic factors is shifted to the angiogenic tendency [4,5,6]. Therefore, downregulation of angiogenic factors or upregulation of anti-angiogenic factors could be tried to prevent neovascularization [4,5,6]. Among these, VEGFA and VEGFR-2 are known to be key factors for the progression of CNV, so numerous studies have been conducted for targeting these factors [4,5,6,7].

Bevacizumab, a recombinant monoclonal immunoglobulin G1 antibody directed against all isoforms of VEFGA, is most commonly used as an anti-VEGF agent for the treatment of CNV [6]. Various routes of administration including topical instillation, intrastromal injection, and subconjunctival injection have been attempted [6,10,12,14,15]. Several clinical and experimental studies have demonstrated that bevacizumab is effective in small-to-medium sized vessels, which have been recently developed [10,13,33]. However, treatment with bevacizumab has several limitations, including the requirement for multiple injections due to its short duration of action, disruption of wound healing leading to stromal thinning, and the paradoxical upregulation of other angiogenetic factors, all of which have been clinically reported [4,6,14,34,35,36]. Hence, some studies have suggested the use of combination therapy comprising bevacizumab and other therapeutics such as corticosteroid, immunosuppressive agent, and laser therapy, including photodynamic therapy, for occluding vessels [11,33,37,38,39].

Rivoceranib has been approved in China for use as a single or combination therapy for various end-stage solid cancers including stomach, colorectal, liver, and adenoid cyst carcinoma [18,20,22]. This small-molecule receptor TKI can block the phosphorylation of VEGFR-2 by binding its intracellular adenosine triphosphate sites [18,19,20]. Hence, rivoceranib can restrain several signaling pathways as follows: the Raf/MEK/Erk pathway, which results in the proliferation of endothelial cells; the p38-MAPK pathway, which stimulates the migration of endothelial cells; and the PI3K/AKT/mTOR pathway, which enhances vascular permeability [18,19,20]. Recently, many studies have shown that rivoceranib could directly act on the process of inhibiting the PI3K/AKT signaling pathway associated with other VEGF-independent compensatory mechanisms [19,21]. At a high concentration, rivoceranib also inhibits c-Kit and c-SRC tyrosine kinases [18,19]. In addition, rivoceranib could directly inhibit the drug efflux transporter including adenosine triphosphate (ATP) binding cassette subfamily B member 1 or ATP binding cassette subfamily G member 2 related to drug resistance [19].

In the present study, we investigated the efficacy of rivoceranib eyedrops by comparing with that of conventionally used bevacizumab solution in a murine model of CNV. The results of the study demonstrate that topical application of rivoceranib can reduce the clinically measured CNV area and index. In addition, the reduction in blood and lymphatic angiogenesis following the topical application of rivoceranib was histologically equivalent to that after the topical administration of bevacizumab. No significant side effects were observed during the experiments. 

Most studies on TKIs used to treat CNV have focused on multi-target TKIs that target VEGFR-1/3 and PDGF as well as VEGFR-2 [14,24,25,26,27,28]. Sunitinib had about three times more efficacy than bevacizumab in an animal study, but side-effects, including iris pigmentation, were also reported [14]. Pazopanib, another multi-target TKI, has been shown to be a safe and alternative therapy for CNV when used topically in human subjects [28]. However, studies for CNV using selective TKI targeted on VEGFR-2 are very rare. In addition, there have been no studies using selective TKI compared to a positive control for treatment of CNV. One study reported that SU5416, an unapproved selective TKI-targeting VEGFR-2, reduces the parameters of CNV compared with those of the untreated group [40]. Lee et al. have shown that rivoceranib, as a loaded human serum albumin-conjugated polyethylene glycol nanoparticle, was more effective from a pharmacologic perspective in treating ocular neovascularization both in vivo and in vitro, compared with the solution form of rivoceranib [23]. 

The limitation of our study is that the effect of topical rivoceranib on corneal inflammation was not evaluated. Anti-VEGF therapies, including TKIs, are known to be more effective in inhibiting angiogenesis from an oncological aspect when combined with other agents which have a different mechanism of action [36,41]. Additional studies may be necessary for evaluating the efficacy of the combination treatment of topical rivoceranib and other therapeutics for the treatment of CNV. 

## 5. Conclusions

To the best of our knowledge, this study is the first to investigate the efficacy of VEGFR-2-selective TKI in comparison to that of topical bevacizumab, the conventional anti-VEGF treatment. Our results suggest that topical application of selective VEGFR-2 TKIs has similar efficacy in inhibiting CNV compared with VEGF-A antibodies. Therefore, topical rivoceranib can serve as a potential therapeutic agent for treatment of CNV.
